# Reclaiming ʻĀina Health in Waimānalo

**DOI:** 10.3390/ijerph17145066

**Published:** 2020-07-14

**Authors:** LeShay Keli‘iholokai, Samantha Keaulana, Mapuana C. K. Antonio, Ikaika Rogerson, Kirk Deitschman, Joseph Awa Kamai, Luana Albinio, Kilauea Wilson, Dawn Kepa, Kuaiwi Laka Makua, J. Kahaulahilahi Vegas, Jane J. Chung-Do, Kenneth Ho, H. Ilima Ho-Lastimosa

**Affiliations:** 1 Ke Kula Nui O Waimānalo, Waimānalo, HI 96795, USA; leshay@hawaii.edu (L.K.); ikaikarogerson@hotmail.com (I.R.); kdeitsch95@gmail.com (K.D.); chungjae@hawaii.edu (J.J.C.-D.); ilima888@gmail.com (H.I.H.-L.); 2 Waimānalo Limu Hui, Waimānalo, HI 96795, USA; awakamai@gmail.com (J.A.K.); waimanalolimuhui@gmail.com (L.A.); waimanaloponoresearchhui@gmail.com (D.K.); 3 Waimānalo Pono Research Hui, Waimānalo, HI 96795, USA; sherrera@hawaii.edu (S.K.); jetney@hawaii.edu (J.K.V.); 4 God’s Country Waimānalo, Waimānalo, HI 96795, USA; 5 Waimānalo Community, Waimānalo, HI 96795; volcanoandsons@gmail.com; 6 Office of Public Health Studies, University of Hawaii at Mānoa, Honolulu, HI 96822, USA; kuaiwi@hawaii.edu; 7 Native Hawaiian and Indigenous Health, Office of Public Health Studies, University of Hawaii at Mānoa, Honolulu, HI 96822, USA; 8 Department of Human Nutrition, Food and Animal Sciences, College of Tropical Agriculture and Human Resources, University of Hawaiʻi at Mānoa, Honolulu, HI 96822, USA; 9 Rossier School of Education, University of Southern California, Los Angeles, CA 90089, USA; 10 College of Tropical Agriculture and Human Resources, University of Hawaii at Mānoa, Honolulu, HI 96822, USA

**Keywords:** native Hawaiian, indigenous, health, land, environment, community-based, qualitative

## Abstract

Kānaka Maoli (Native Hawaiian) worldviews of health emphasize pono (righteousness) and lōkahi (balance), which extends to include relationships with other people, akua (spiritual realm), and ʻāina (land). The purpose of this qualitative study was to explore the role of ʻāina and ʻāina connection in health and resilience based on the perspectives of 12 Kānaka Maoli adults from the Waimānalo community. Three major themes were identified: ʻĀina is everything, ʻāina is health, and community healing through community-led initiatives. A better understanding of ʻāina connection is important to improve our knowledge of Hawaiian health. A connection to ʻāina may specifically address health concerns resulting from historical trauma and environmental changes.

## 1. Introduction

Kānaka Maoli (Native Hawaiian; literally Indigenous people; used interchangeably with Kānaka) health encompasses more than physical and mental well-being. Respected kūpuna (elders), Paglinawan and Paglinawan [1], and Hawaiian worldviews understand health to include the balance and well-being of spirituality, land, and people [2]. In addition, Kānaka Maoli health includes wellness of spiritual, physical, and mental health. An imbalance between the previously mentioned entities can cause sickness in an individual. Likewise, an imbalance between spirituality, land, and people can also cause sickness in individuals, families, and communities. For instance, the Kūkulu Kumuhana framework [3] acknowledges Kānaka well-being to encompass various domains, namely ʻāina momona, or healthy and productive land and people. This framework was adapted from the social ecological model [4] and encompasses Kānaka identity, multi-dimensionality, social justice, and holism [3]. It recognizes the following concepts as the core of Kānaka Maoli well-being: Ea (self-determination), ʻāina momona (healthy and productive land and people), pilina (mutually sustaining relationships), waiwai (ancestral abundance, collective wealth), ke akua mana (spirituality and the sacredness of mana), and ʻōiwi (cultural identity and native intelligence). All six concepts span across the following dimensions: ʻOhana, community, organization, and policy.

Similar to other Indigenous communities [5,6], colonization, historical trauma, and control of political institutions by settlers in Hawaiʻi changed the way medical and public health professionals addressed health [7,8]. Western models and ways of life were adopted, excluding Kānaka concepts of health and well-being from Kānaka lifestyles and care. Traditional ways of life became inaccessible. For example, the familial and reciprocal relationship between land and people was challenged when land privatization policies entered the Hawaiian Kingdom in 1848 [9]. Unfamiliar with the concept of land ownership, Kānaka claimed less than 1% of Hawaiian lands [10]. As a result, many Kānaka lost access to the lands they tenured, which caused a disconnection with their food supply, economic self-sufficiency, families, communities, and well-being. Repercussions of land privatization and the myriad of traumatic historical and political events associated with colonization are still ongoing and have severely impacted the health, social, and economic status of present-day Kānaka. For example, Kānaka Maoli experience more homelessness and houselessness than other major ethnicities in Hawaiʻi [11]. They are at higher risk for chronic diseases and substance use [12,13,14]. In addition, they are disproportionately represented in the incarcerated population and experience domestic violence at higher rates than other major ethnicities in the state [15,16].

Despite the increased exposure to historical trauma and adversity, Kānaka Maoli have persevered and are now one of the fastest growing populations in the nation with Asian Americans and Other Pacific Islanders [17]. Like other Indigenous peoples, Kānaka Maoli demonstrate resilience [18,19] and continue to thrive while enduring the long-standing impacts of historical trauma. One way Kānaka Maoli continue to demonstrate their resilience is through their ability to sustain a connection to ʻāina. Based on Kānaka Maoli ways of knowing, ʻāina is an indicator of health and wellness [2,3]. Therefore, the purpose of this qualitative study was to explore the role of ʻāina and ʻāina connection in health and resilience based on the perspectives of 12 Kānaka Maoli adults from the Waimānalo community, a rural town on the island of Oʻahu.

### 1.1. The Role of ʻĀina

Similar to other Indigenous Peoples, the origin story of Kānaka Maoli illustrates the reciprocal relationship between land and people. In the moʻolelo (story) of Hāloa, Wākea (sky father) and Hoʻohōkūkalani fall in love and have a stillborn child whom they bury. Out of the burial, births the first kalo (taro) plant, which they name Hāloanakalaukapalili. When they become pregnant again, Hoʻohōkūkalani births a baby boy named Hāloa in honor of his older brother, the kalo plant. Hāloanakalaukapalili cares for his younger brother by providing sustenance, while Hāloa serves his older brother through mālama ʻāina (land stewardship, care for the land). An ʻōlelo noʻeau, a Hawaiian proverb, demonstrates this relationship. He aliʻi ka ʻāina; he kauā ke kanaka [20]. The land is chief, people are its servant. The Hawaiian people value land stewardship, as demonstrated in their moʻolelo, teachings, and practices.

While these teachings continue to be preserved and passed on today, contemporary Hawaiians must heed to the requirements of survival, which include working away from their ancestral lands in careers that may not align with their values as Hawaiians, but afford an opportunity to live comfortably in a western world. Despite these adversities, Kānaka continue to remain resilient as demonstrated through the revitalization, resurgence, and perpetuation of cultural practices and ways of being, including practices that allow for reciprocity and connections to ʻāina [21]. Cultural protocols from ancient knowledge have been revitalized in the last few decades and are now a lifestyle for many Kānaka, which reinforces alignment with all elements.

### 1.2. Context of Study and the Waimānalo Community

Waimānalo is a rural town on the windward coast of Oʻahu, Hawaiʻi, with nearly one third of the residents being Kānaka Maoli or Pacific Islander alone [22,23]. Although Waimānalo is considered a community of high socio-economic need with educational and health disparities [23], it boasts many strengths and serves as a hotspot for community advocacy, cultural revitalization, resource restoration, and sacred site restoration. For instance, Waimānalo is home to various community initiatives such as Waimānalo Pono Research Hui and the Waimānalo Limu Hui.

The Waimānalo Pono Research Hui serves as an equivalent to a community advisory board and/or community research review board [24,25]. As a hui (group, organization) of community and academic partners, members of the Waimānalo Pono Research Hui identified three priority areas to address through health action and research. Limu (plants living under water) restoration was among the three priority areas. This led to the development of Waimānalo Limu Hui, an organization established in Waimānalo, Oʻahu in November 2017, with a focus on limu restoration, primarily in the Waimānalo community. Their mission is to “provide sustenance, food, and medicine for the Waimānalo community while improving the ecosystem in perpetuity” [26]. As a result of this identified priority area, Waimānalo community members quickly assembled to learn from Kua ʻĀina Ulu ʻAuamo (KUA)’s Limu Hui Coordinator, Uncle Wally Ito. Since then, Waimānalo Limu Hui has held monthly limu planting days and Pāhonu (an ancient loko iʻa or fishpond for sea turtles) restoration days every three months. According to a recent evaluation, 95% of people who participated in the Waimānalo Limu Hui day found the experience to be unforgettable, 91% identified the community engagement, and, most importantly, 97% indicated this was a form of aloha ʻāina, or a deep love and connection for the land [27].

### 1.3. Purpose of Study

This study expands on our previous research, which explored the concept of health as being holistic and the concept of resilience as a multi-dimensional construct [28]. In our previous study, we qualitatively explored conceptualizations of health and resilience among Kānaka Maoli based on 12 residents of Hawaiian Homestead Lands throughout Hawaiʻi (i.e., on the islands of Oʻahu, Molokaʻi, and Hawaiʻi Island) in 2016–2017. Findings from our previous study identified four major themes including: Health maintained through balance, being unhealthy vs. being ill, the concept of colonialism and resulting adversities, and protective and resilience factors that foster health. It was determined that cultural values and cultural practices may help to address health inequities experienced by Kānaka Maoli, and in particular, a connection to ʻāina is a prominent factor of health and resilience for Kānaka Maoli. Similar health action and research priorities have been identified in the Waimānalo community. Therefore, the purpose of this study was to explore the role of ʻāina and ʻāina connection in health and resilience based on the perspective of Kānaka Maoli residing in the Waimānalo community using qualitative methods.

## 2. Materials and Methods

### 2.1. Participants

This study implemented seven key informant interviews and two small focus groups (*n* = 2–3 per focus group) to qualitatively explore the concept of ʻāina in relation to health and resilience from the perspective of Kānaka Maoli adults. Key informant interviews and focus groups were conducted with Kānaka Maoli community members/leaders (i.e., cultural practitioners and those involved in ʻāina-based programs) from the Waimānalo community. Purposive and convenience sampling strategies were employed. Purposive sampling allows for diversity in characteristics among participants. Convenience sampling strategies were also employed as the interviews took place during a community-organized event including Waimānalo Limu Hui community days and Waimānalo Pono Research Hui meetings. Interviews were conducted between 2019–2020.

### 2.2. Measures

Based on the existing literature and consultation provided by community leaders and experts in the field, a semi-structured guide was created. The semi-structured guide included questions such as, “What does ʻāina mean to you?”, “How would you know if someone is connected to ʻāina?”, and “How is ʻāina related to health?” Probing questions were asked during the interview to gain additional insight on the person’s perspective of the relationship between ʻāina, health, and resilience.

### 2.3. Procedures and Data Analysis

This study utilized community-engaged approaches, such as community-based participatory research (CBPR) and ʻĀina Aloha Research Frameworks to assess perceptions of ʻāina and ʻāina connection in relation to health and resilience [29,30,31,32,33,34]. The CBPR approach to research included community members in all phases of the study, including co-authorship in this manuscript. The success of this study, including successful implementation of CBPR approaches, which prioritized community needs and priorities, is attributed to the culmination of community partners including the Waimānalo Limu Hui and Waimānalo Pono Research Hui, a doctoral research project funded through the Kūlana Noiʻi Award, and a larger pilot project, entitled *Ke ola o ka ʻāina. The role of ʻāina connectedness in Kānaka health,* a pilot project funded through Ola HAWAII (project number 2U54MD007601-31). The doctoral research project and larger pilot project were supported by existing partnerships between the doctoral researcher, the University of Hawaiʻi at Mānoa faculty mentors, Waimānalo Pono Research Hui members, and Waimānalo Limu Hui.

The research team and Waimānalo Limu Hui are part of the Waimānalo Pono Research Hui, a community and academic partnership that aims to achieve health through pono research by the community and for the community. The lead author (L.K.) holds a leadership role in the Waimānalo Limu Hui and has been actively involved in the Waimānalo Pono Research Hui since its inception. The recipient of the doctoral research project (S.K.) has been a part of Waimānalo Pono Research Hui since its conception in February 2017. In 2018, the doctoral researcher co-developed Pono Research Protocols and Rules of Engagement, which outlines the academic and community partnership and specific principles of engaging in research to ensure ethical and pono research in the Waimānalo community [22]. Through this process, the Waimānalo Pono Research Hui Shark Tank was developed, which is equivalent to a community internal review board application.

The faculty mentor (M.C.K.A.) for the doctoral research project and the principal investigator of the project, *Ke ola o ka ʻāina. The role of ʻāina connectedness in Kānaka health*, also has established relationships with Waimānalo Limu Hui members and has gone through a rigorous screening process to partner with the Waimānalo Pono Research Hui to conduct this research study in Waimānalo. Through this process, it was determined that the Waimānalo community owns all data in this study, including the qualitative stories that were shared during this process. Negotiations related to data management were an important part of the process to ensure data ownership and data sovereignty among the Waimānalo and Kānaka Maoli community. Similar to other Indigenous communities, research may have a negative connotation to Kānaka Maoli communities, which has led to a sense of mistrust of the research community [35]. Data sovereignty helps to bridge the gap of mistrust by allowing communities to have full control over data management and ownership. Data sovereignty may help to protect intellectual property of these communities, the dissemination of community and intergenerational knowledge, while allowing for Indigenous self-determination [36,37].

Community experts and key Waimānalo Limu Hui members provided support around recruitment and ensured the community was involved in all stages of the research process. Interviewees were selected based on their involvement in ʻāina-based programs, ʻāina activism, or due to their role as a Kānaka Maoli cultural expert/practitioner. Community experts included leaders of ʻāina-based programs and other initiatives in the Waimānalo community. They were also identified by participants as a community leader who played a critical role in intergenerational knowledge and community preservation of ʻāina or ʻāina-based activities. Community experts helped to co-develop qualitative study questions, co-analyze, and provide support in dissemination. This study was also approved by the university IRB.

The seven key informant interviews comprised one interviewee, while one focus group comprised two interviewees, and one focus group comprised three interviewees. Interviews and focus groups were conducted by the second and third authors. The interviews and focus groups ranged in duration from 30 to 60 min and took place before, during, or after a community-organized event. Interviewees consented to participate in this study and to be audio recorded, which allowed for transcription of interviews verbatim. Interviewees were also thanked for their time and were compensated with a $25 gift card. Transcriptions were analyzed using a grounded theory approach [38]. The three lead authors of this study immersed themselves in all of the audios and audio transcriptions of each interview and focus group. Each transcription was reviewed separately (i.e., each key informant interview was analyzed as its own transcript and each focus group was considered as its own transcript). Next, the three lead authors reviewed one of the focus groups collectively, to allow for the creation of a codebook. The remaining interviews and focus group were then reviewed independently by the three lead authors and later reviewed by the group. In the case of disagreement, the three researchers determined coding based on group consensus. In alignment with Kānaka ways of knowing, the research team acknowledged that many of the themes are interconnected with one another. Therefore, the most salient and recurring codes were classified as a theme.

## 3. Results

### 3.1. Participant Characteristics

A total of 12 interviewees were recruited and interviewed for this study. Of the 12 interviewees, 5 (42%) were female and 8 (67%) were kūpuna (elders). Most of the interviewees were either married (*n* = 4, 33%) or divorced, separated, or widowed (*n* = 5, 42%). All of the participants resided in the Waimānalo community. A summary of the participant characteristics is provided in Table 1 (below).

### 3.2. Summary of Themes

Using a grounded theory approach, three major themes were identified: (1) ʻĀina is everything, and therefore, we as people are ʻāina, (2) ʻāina is health, and (3) community healing through community-led and ʻāina-based initiatives. Each theme comprised the following subthemes: (1) Connection, (2) intergenerational knowledge, (3) colonization, and (4) mālama. Each theme is described in greater detail below. Table 2 provides a list of the three themes with a codebook definition and direct quotes to highlight each theme.

### 3.3. Theme 1: ʻĀina is Everything; therefore, We as People are ʻĀina.

“ʻĀina is everything”; therefore, we as people are ʻāina was a prominent theme. As one of the interviewees put it, “[ʻĀina means] everything. It’s all connected on every level mentally, spiritually, physically... not just land, not just ocean... everything.” Collectively, interviewees described ʻāina as the land, the ocean, the sea, all the elements, plants, people, and their interconnection. Interviewees indicated they were a part of ʻāina and that there is no separating ʻāina from Kānaka. While describing ʻāina, four sub-themes emerged, which included: Connection, intergenerational knowledge, colonization, and mālama.

Interviewees indicated that the connection between ʻāina and Kānaka is spiritual and reciprocal. They described ʻāina as sustenance, that which feeds Kānaka physically, spiritually, mentally, and emotionally. Therefore, a reciprocal relationship with ʻāina is pivotal to maintaining survival and connection. The connection to ʻāina is important to Kānaka health and stems from their genealogy, respect for ʻāina, and kuleana (loosely translated as responsibility) to care for and intimately engage with ʻāina. Interviewees described the intimacy between people and the land as familial connection and related this connection to the story of Hāloa (refer to the introduction for additional information about the story of Hāloa). From this moʻolelo, interviewees expressed that people were tasked forever to care for kalo, in essence ʻāina, as kalo and ʻāina would provide sustenance for Kānaka.

In addition to ʻāina providing food for Kānaka, kūpuna interviewees described how ʻāina is medicinal, as doctors were not readily available in Waimānalo like in urban areas of Oʻahu. According to one of the interviewees, “You hardly see people going to the doctor in Waimānalo. I mean never have doctor. Was too far.” Therefore, parents and grandparents taught interviewees to primarily rely on ʻāina through identifying, picking, and preparing home remedies, like lāʻau lapaʻau (Hawaiian traditional medicine) and cleansing through hiʻuwai (water purification festivity where people bathe in the sea or stream). Another interviewee expressed this point through the following quote, “Our parents would treat us with homemade remedies... they took care of us with what they had, and they understood the plants and how to use them.” Intergenerational health was reflected through intergenerational knowledge about ʻāina passed on from parents, grandparents, kūpuna, and through a sense of kuleana to pass this knowledge on to future generations. Participants described this kuleana of caring for ʻāina as a way of caring for the land, themselves, their communities, and their keiki (children).

Interviewees described the difficulties that disrupted the values that maintained the familial connection between land and Kānaka. They related the difficulties to the impacts of colonization, where they or their parents were forced to abandon their intimacy with ʻāina to find work that would provide monetary sustenance and help Hawaiian families survive in a changing Hawaiʻi. That work pushed them into positions outside of Waimānalo, away from ʻāina. The losses suffered resulted in intergenerational trauma, where Waimānalo started to experience homelessness, substance abuse, and other difficult issues. Because of this hurt, interviewees described Waimānalo Limu Hui’s limu restoration effort taken place by the Waimānalo Limu Hui, with tears, affection, and deep appreciation. Initiatives such as the Waimānalo Limu Hui illustrated to interviewees that multiple truths and worldviews exist. Moreover, while the impacts of colonization disrupted the values and connection to ʻāina, some maintained those values and their connection to ʻāina. Presently, the connectedness to ʻāina is being revitalized and realized through community initiatives like the Waimānalo Limu Hui.

Among the values and practices being revitalized to promote connection to ʻāina was mālama, or to care for, ʻāina. Interviewees depicted mālama ʻāina as a practice that reciprocates the multitude of benefits provided by ʻāina. Keeping ʻāina clean and presentable were identified as a reflection of the physical, mental, spiritual, and emotional health of Kānaka. Clean ʻāina meant clean food and a healthy body, mind, and spirit. Health of self by mālama ʻāina extended to the intergenerational healing from colonization among Kānaka and ʻāina. Interviewees indicated that healing the trauma passed down from previous generations happens by bringing family and community members back together, and encouraging them to practice mālama ʻāina. They identified building the wall at Pāhonu, planting limu with the Waimānalo Limu Hui, or growing their own food and lāʻau lapaʻau in their backyard aquaponics systems as the mālama ʻāina practices pivotal in their own healing. These activities also allow kūpuna and makua (adults) to demonstrate and pass down ʻike kūpuna (ancestral knowledge), and cultural knowledge and practices aligned with mālama ʻāina.

### 3.4. Theme 2: ʻĀina is Health.

The overall theme of ʻāina is health focused on ʻāina as an interconnected part of overall health and well-being. In alignment with the previous theme, the four sub-themes within this larger theme included: Connection, intergenerational knowledge, colonization, and mālama. A connection to ʻāina was demonstrated through people opening their hearts and acknowledging the healing properties of ʻāina. These healing properties were not only physically healing, but the interviewees also identified an emotional and spiritual healing. As one of the interviewees put it, “We think it’s just physical healing, but there’s that spiritual one, that emotional connection. Once you get that connection with ʻāina you’re already feeling better. To me, mental and spiritual health manifests spiritually. If we [are] not taking care of all aspects, then of course we’ll be unbalanced and get sick.” When interviewees and the general community allowed ʻāina, personified as “him” or “her”, into their heart, ʻāina allowed for a “gentle and kind healing”. Healthy ʻāina and a healthy connection to ʻāina was a clear indication of the health of Kānaka, or people. Therefore, one can innately tell when a person was not connected to ʻāina and when the person did not mālama (take care of) ʻāina.

Intergenerational knowledge of ʻāina as health was indicated by knowledge that has been learned and passed down over time from ʻohana (family), kūpuna (elders, including ʻāina as an elder), and leaders in the community. Lāʻau and the ocean were the most frequently cited sources of ʻāina is health in the form of intergenerational knowledge. When one of the interviewees referred to the ocean, they pointed out that, “To me, this was our healing. Healing soul.” Another interviewee noted, “When we were sick, we went down to the ocean for healing. We didn’t have the runoff or the chemicals that come down from golf courses and people using chemicals on the land with farming.” Lāʻau was seen as any type of plant that was grown in or on ʻāina. Lāʻau could also be used as foods for medicinal plants that helped to facilitate and maintain health. The focus of lāʻau was particularly salient for those who identified as a cultural practitioner. The ocean was also referenced as having healing properties with knowledge of the ocean being seen as intergenerational knowledge. The importance of the ocean having healing properties was commonly cited during the Waimānalo Limu Hui planting days, in which the ocean allowed for healing and community building.

Colonization was cited as a significant factor that resulted in negative impacts from outsiders and foreigners that led to attacks and desecration of ʻāina, and therefore, health. Prior to colonization, ʻāina was seen as being healthier, abundant, and plentiful. According to one of the interviewees, “We grew up learning how to swim here at Pāhonu. Our uncles use [*sic*] to take us fishing and spear diving... it was plentiful... now it’s scarce. No more that much and it was [gone] in a short time.”

Negative consequences of colonization included a large disconnection to ʻāina, which created unbalanced health, manifesting as physical, mental, emotional, and spiritual sickness. Despite the negative consequences of colonization, mālama was the last sub-theme that identified the way Kānaka have a kuleana to mālama the ʻāina. All of the interviewees discussed the importance of mālama ʻāina, or taking care of the ʻāina, with the understanding that ʻāina will always take care of us. By engaging in mālama ʻāina, ʻāina not only heals us, but ʻāina allows for intergenerational healing. The discussion related to intergenerational healing is further discussed below in Theme 3.

### 3.5. Theme 3: Community Healing—Resulting from Community Initiatives

As described in Themes 1 and 2, impacts of colonization caused a disruption of values and connection to ʻāina among interviewees. Realizing the benefits of health through reconnecting to ʻāina practices and traditional values, community healing as a result of community initiatives was a salient theme. Following suit, four sub-themes transpired: Colonization, connection, intergenerational knowledge, and mālama.

Colonization was described as an attempt of genocide among the Hawaiian people, which is presently reconciled with community healing through connection and mālama ʻāina. The Great Māhele (the official political event that divided lands for ownership; prior to, land was not owned, rather land was traditionally stewarded by its people) was indicated as an example of disruption, particularly with the Hawaiian worldview of land tenureship, where Hawaiians were assimilated to western values and foreign practices, such as land ownership. Interviewees suggested that the invasion on Kānaka by westerners and missionaries forced and imposed policies/events, like the Great Māhele, and stripped Kānaka of their lands, language, culture, and values to make them reliant on western settlers, their governments/organizations, and their ways of life. They also influenced the relationships between community members, causing distrust and trauma that has been passed down to present-day generations. However, while the impacts of this attempted genocide were threatening and hurtful, interviewees suggested ʻāina as the solution to overturn the trauma.

Community initiatives were believed to be restoring the connection to ʻāina and through the work of aloha ʻāina is aloha (loosely translated as love, affection, compassion). The aloha of ʻāina was apparent in the strain of interviewees’ voices. They explained that community initiatives, like limu restoration, have given them the ability to restore and mālama the relationships with themselves, their family members, community members, and the land. By participating in community initiatives, they are able to be intentional about what they manifest with ʻāina. Clean thoughts, care, and aloha put into the ʻāina by Kānaka were mentioned as pertinent to receiving clean and nutritious sustenance from ʻāina.

In addition to restoration of aloha ʻāina (love for ʻāina), community initiatives have sparked the sharing of intergenerational knowledge. During community initiative events, kūpuna have shared traditional place names and moʻolelo (stories) with younger generations, which interviewees indicate is important to Hawaiian identity and reconciling the impacts of colonization. Community initiatives promote the values of giving, sharing, and mālama. They have been able to connect with more people in their community and upkeep these relationships through sharing knowledge, resources, lāʻau lapaʻau recipes, and organization of community events.

The value of mālama ʻāina was significant in community healing. Interviewees cited being healed by ʻāina by working on Pāhonu and replanting limu with the Waimānalo Limu Hui. Kūpuna interviewees expressed their deep appreciation for young people who put their hands into Pāhonu, promote cultural identity, and share traditional values and practices. In addition, community initiatives created a foundation for interviewees to practice sharing and giving away of resources, aloha, and knowledge. They identified these practices as a means to make their hearts and naʻau (guts) feel good. Mālama ʻāina through community initiatives was understood to be a replica of the past and a solution to the historical and intergenerational trauma suffered by their grandparents, parents, and themselves.

## 4. Discussion

The primary purpose of this study was to explore the role of ʻāina and connections to ʻāina in health and resilience based on the perspective of Kānaka adults from the Waimānalo community using qualitative methods. Findings from this study emphasize the connection between ʻāina and Kānaka. Entities of akua (spiritual realm), ʻāina, and Kānaka are intrinsically linked, creating pilina (mutually sustaining relationships), which allows for each entity to thrive through synergy and interdependence. Balance and harmony between the three entities cultivates optimal health. Imbalances, such as disconnection between ʻāina and Kānaka, creates physical, spiritual, emotional, and mental maʻi (sickness) in Kānaka Maoli, indicating that ʻāina is essential to the health of Kānaka.

Findings align with previous research that emphasize the importance of ʻāina as an indicator of health [28,39,40]. In particular, ʻāina and a connection to ʻāina is a key solution to health and addressing health concerns that have stemmed from colonization and social injustices [41]. Reconnecting with ancestral land and cultural practices has been shown to address health inequities and promote resilience among other Indigenous communities [42,43]. Stronger relationships and continued connections to ʻāina is a demonstration of Hawaiian values and the resilience of the Hawaiian community despite the negative consequences of colonization, historical trauma, and historical loss. The role of ʻāina may specifically address health concerns related to sickness that have resulted from disconnection to ʻāina, colonization, historical trauma, and environmental changes by fostering a stronger connection to ʻāina. This is particularly demonstrated through the perceptions of ʻāina as being everything and as having healing properties.

One significant part of the research process that is important to acknowledge is the Indigenous approach to research. The success of this study resulted from the multiple stakeholders involved in the study and the relationships between the community and the research team. The CBPR approach supplemented with ʻĀina Aloha frameworks enhanced the success of this study. Taking a community-based and Indigenized approach to research has also been shown to be effective in other Indigenous communities [29,30,31,32,33,34,35,44]. The setting of the research study also played an important role in the interview process, as it allowed interviewees to feel comfortable and grounded while engaging in interviews about ʻāina. Furthermore, the research team comprised members from the Waimānalo community, members from the Kānaka Maoli community, and/or those who have been actively engaged in research and service in the Waimānalo community. These relationships helped to facilitate the partnerships and successes of this study.

Theoretical saturation was achieved due to consistencies in responses across interviewees. There were also no significant differences in thematic development based on the qualitative method employed including key informant interviews and small focus groups. Despite the strengths of this study and achieving theoretical saturation, limitations must be acknowledged. Firstly, the findings from this study may not be generalizable to other Kānaka Maoli adults or communities. Furthermore, although community members were involved in the entire research process, the findings from this study may still be limited to the interpretations of the research team and those involved in the research process. As a result, future research may expand on this study by including other Kānaka Maoli and communities who also identify ʻāina, ʻāina connection, and ʻāina health as a community research and action priority. Similarly, although a grounded theory approach was used to analyze data, a semi-structured interview guide was created a priori. Furthermore, although participant quotes were provided throughout the manuscript to demonstrate codebook examples and definitions based on audio recordings that were transcribed verbatim, the development of themes were based on the interpretations of the research team and did not use participant language in vivo. To account for this limitation, the interview guide continued to evolve based on the ongoing feedback provided by community leaders and interviewees.

## 5. Conclusions

There is a pressing need to address health inequities experienced by Indigenous Peoples globally, including Kānaka Maoli, the Indigenous Peoples of Hawaiʻi [5,6,12,13,14]. Findings from this study continue to emphasize the importance of integrating holistic practices that include ʻāina-based connections and healing as a mechanism of bettering the health of Kānaka Maoli [26]. Connections with ʻāina will particularly aid in addressing health inequities experienced by Kānaka Maoli that stem from colonization, that have increased displacement and disconnections to ʻāina. Holistic practices that integrate ʻāina-based connections and healing will also help to strengthen relationships between ʻāina momona (healthy and productive land and people) and other core concepts of health based on Kānaka Maoli ways of knowing, including waiwai (ancestral abundance, collective wealth), ke akua mana (spirituality and the sacredness of mana), and ʻōiwi (cultural identity and native intelligence) [3]. This will ultimately promote the general health and well-being of Kānaka Maoli, thereby leading to ea (self-determination), allowing Kānaka Maoli to identify their own health priorities and needs, while remaining resilient in their ancestral homelands.

While limitations exist, this study has implications for future research and practice. To begin, this study has implications for future research by demonstrating the importance of building rapport and a relationship when engaging in research, identifying community health priorities as part of the research process, and having the community involved in all aspects of the research process. The findings from this study also have implications for future practice that focus on Kānaka health. This study demonstrates the importance of considering ʻāina as a significant factor of Kānaka health. A holistic approach to health should also consider the role of culture, spirituality, kūpuna (elder) knowledge, and ʻohana (family) in relation to ʻāina and health. Findings from this study also demonstrate the urgent need to stand with Indigenous peoples who continue to resist policies and structural forces that threaten their existence and Indigenous lands. Therefore, these findings have significant implications for future research, policies, and interventions that strengthen resilience and address health of Kānaka Maoli by considering the role of ʻāina in health.

## Figures and Tables

**Table 1:** Characteristics of key informant interview participants.

Characteristics	Values *n* (%)
Gender	
Male	7 (58%)
Female	5 (42%)
Ages	
Mākua (35–54 years)	4 (33%)
Kūpuna (55 and older)	8 (67%)
Marital status	
Single or in a relationship but not married	3 (25%)
Married	4 (33%)
Divorced, separated, or widowed	5 (42%)
Cultural practitioner	8 (67%)

**Table 2:** Summary of themes.

Theme	Definition	Examples (Quotes)
**Theme 1:** ʻĀina is everything, and therefore, we as people are ʻāina.	Code as theme if the information was interpreted as the following: 1. **Connection:** What feeds us (not just physically, spiritually, mentally emotionally), we are all connected.	1. **Connection:** “[ʻĀina means] everything. It’s all connected on every level mentally, spiritually, physically... not just land, not just ocean... everything.” “ʻĀina is everything. It holds us. It keeps us. ʻĀina is the keeper, we [are] just part of it.”
	2. **Intergenerational Knowledge:** Intergenerational health is reflected through intergenerational knowledge about ʻāina passed on from parents, grandparents, kūpuna, and through a sense of kuleana to pass this knowledge on to future generations.	2. **Intergenerational Knowledge:** “Our parents would treat us with homemade remedies... they took care of us with what they had and they understood the plants and how to use them.”
	3. **Colonization:** Disrupted values and integrated intergenerational knowledge with intergenerational trauma, especially in relation to people’s connection with ʻāina. Colonization also led to an acknowledgement that multiple truths exist.	3. **Colonization:** “Everybody’s connected to a thing [technology] instead of connected to each other.” “As we got older, they developed frozen foods and convenience foods. So we began to change and eat those things which made us very momona and unhealthy… what we need to do is go back to eating… the foods that we grew up with then the health will return.” “Eating ʻāina-based foods is hard too because we no more ʻāina to grow.” “Nobody owns the land. That was the mentality... It’s everybody’s as long as you take on the kuleana. It’s your kuleana, but it’s not your land... we cannot technically own any land.
	4. **Mālama:** Acts of reciprocity for ʻāina, a family member. Mālama also led to intergenerational healing with community and family activities lefted in mālama ʻāina.	4. **Mālama:** “The quality of the land and the water is a reflection of the quality of our health as people.”
**Theme 2:** ʻĀina is health.	Code as theme if the information was interpreted as the following: 1. **Connection:** When people open up their heart, ʻāina will heal. Healthy connection to ʻāina leads to healthy people.	1. **Connection:** “We think it’s just physical healing, but there’s that spiritual one, that emotional connection. Once you get that connection with ʻāina you’re already feeling better. To me, mental and spiritual health manifests spiritually. If we [are] not taking care of all aspects, then of course we’ll be unbalanced and get sick.” “Nutrition comes directly from the land. Medicines come from the land. Everything comes out of the land, even us, knowing that we come from kalo... so healing comes out of the land, as well as birth comes out of the land.”
	2. **Intergenerational Knowledge:** Lāʻau and ocean as healing and a mechanism of sustaining health.	2. **Intergenerational Knowledge:** “When we were sick, we went down to the ocean for healing. We didn’t have the runoff or the chemicals that come down from golf courses and people using chemicals on the land with farming.”
	3. **Colonization:** Prior to colonization, ʻāina was a lot healthier and plentiful. The negative impacts of outsiders and foreigners also lead to negative impacts on health.	3. **Colonization:** “We grew up learning how to swim here at Pāhonu. Our uncles use to take us fishing and spear diving... it was plentiful... now it’s scarce. No more that much and it was in a short time.”
	4. **Mālama:** Intergenerational healing. We take care of ʻāina because ʻāina takes care of us.	4. **Mālama:** “It’s important to know... ʻāina is yours and take care of it because she’s [ʻāina] gentle, kind, healing. You can always go to her.”
**Theme 3:** Community healing resulting from community initiatives	Code as theme if the information was interpreted as the following: 1. **Colonization:** Traumatic events or adversities from colonization that had severe impacts on the community at large.	1. **Colonization:** “Take away language, culture, to make us reliant. They give us food instead of making us grow our own, making us dependent on the government. They took away what was self-sustaining us. In a perfect world, we need to go back.”“When the haoles made [land ownership]... they went and bought up places and places and the [Hawaiians] didn’t understand. They took all the land away from them... the land that they lived on.”“The cost of living is too high…. Everybody that we know is participating in something that is not working… ʻāina is the solution. We cannot live without it. It’s being manipulated… in the name of money, not in the name of mālama.”
	2. **Connection:**Connection to ʻāina is aloha. This is expressed through relationships and mālama.	2. **Connection:** “I’m so touched by these young people of Waimānalo that they give their all to build Pāhonu and plant this limu. It means everything to me… I’m so grateful to this Limu Hui and everyone connected… it’s life, it’s sustenance to have [limu] come back. This is kānaka living.”
	3. **Intergenerational Knowledge:** Sharing of place names and moʻolelo, and values of giving, sharing, mālama.	3. **Intergenerational Knowledge:** “By learning who we are and where we came from through moʻolelo and our values, it will allow us to move forward and face challenges that come up because we’ll be grounded in ourselves and in our community.”
	4. **Mālama:** Healing happens through mālama ʻāina.	4. **Mālama:** “Many people come to the [Waimānalo] Limu Hui and they feel a part of the solution... you come here, you do something, and you see a physical result from it... teaching all these babies they can do things, they can make a difference.”
